# Carbon Footprint of Fluorinated Gases Used in Endothelial Keratoplasty

**DOI:** 10.1097/ICO.0000000000003707

**Published:** 2024-10-02

**Authors:** Alfredo Borgia, Matteo Airaldi, Stephen Kaye, Vito Romano, Roberto Dell’Omo, Kunal Gadhvi, George Moussa, Raffaele Raimondi

**Affiliations:** * St. Paul's Eye Unit, Liverpool University Hospitals NHS Foundation Trust, Liverpool, United Kingdom;; † Department of Eye and Vision Science, Institute of Life Course and Medical Sciences, University of Liverpool, Liverpool, United Kingdom;; ‡ Department of Molecular and Translational Medicine, University of Brescia, Brescia, Italy;; § Department of Medical and Surgical Specialties, Radiological Sciences, and Public Health, University of Brescia, Brescia, Italy;; ¶ Department of Medicine and Health Sciences, University of Molise, Campobasso, Italy;; ║ Manchester Royal Eye Hospital, Manchester University Hospitals NHS Foundation Trust, Manchester, United Kingdom; and; ** Newcastle Eye Centre, Royal Victoria Infirmary, Newcastle Upon Tyne, United Kingdom.

**Keywords:** fluorinated gases, endothelial keratoplasty, DMEK, environmental impact

## Abstract

**Purpose:**

The purpose of this study was to examine the direct impact on carbon emissions attributed to the use of fluorinated gases in endothelial keratoplasty (EK) procedures using gas tamponade and to evaluate the respective carbon footprint of different gas delivery systems used in EK procedures.

**Methods:**

In this retrospective, single-center environmental impact study, all corneal procedures using fluorinated gases between January 2021 and January 2024 at the Royal Liverpool University Hospital were reviewed and included. The CO_2_ equivalent emissions were calculated based on the mass of each fluorinated gas used, following the guidelines of the Intergovernmental Panel on Climate Change.

**Results:**

Of 357 total procedures (160 Descemet membrane endothelial keratoplasty [44.8%], 118 Descemet stripping automated endothelial keratoplasty [33.1%], and 79 rebubbling [22.1%]), single-use sulfur hexafluoride (SF6) canisters were used in 278 (77.9%) procedures. SF6 canisters used in corneal transplantation emitted nearly 1.5 tons of CO_2_ over 3 years. The 30-mL canisters emitted twice the CO_2_ per GBP compared to SF6 15-mL canisters and 4 times that of C2F6 or C3F8 15-mL canisters.

**Conclusions:**

Fluorinated gas use in corneal transplantation has a significant environmental impact, which can be reduced by the use of smaller single-use canisters with lower carbon footprint, although manufacturing and disposal concerns remain. Our findings advocate for a more environmentally conscious approach to EK, favoring the use of smaller, more cost-efficient canisters and considering air as an alternative tamponade where possible.

Climate change, characterized by a surge in extreme weather events and infectious diseases, is a critical concern for global health and poses significant threats to our planet and its ecosystem.^1^ Attaining the targets set by the Paris Climate Change Agreement could result in significant health benefits, potentially saving more than 5700 lives annually because of better air quality, around 38,000 lives each year through increased physical activity, and over 100,000 lives annually as a result of improved dietary habits.^2^

It is now widely recognized that human actions are the major driving force behind climate change, with health care delivery being a notable example of such activities.^3^ The UK's National Health Service, which contributes 5.4% of the country's emissions, is leading the charge by committing to net zero emissions by 2040, a pioneering move for national health care systems.^4^ Attaining this ambitious target involves recognizing the critical sources of greenhouse gas emissions and developing countermeasures across all areas of health care.

In the realm of intraocular surgery, the use of fluorinated gases such as sulfur hexafluoride (SF6), C2F6, and C3F8, known for their significant greenhouse impact, is widespread.^5^ SF6, in particular, has been targeted for rigorous regulation under the Kyoto Protocol because of its profound global warming effects.^6^ Notably, among fluorinated gases, SF6 emerges as the predominant choice as a tamponade agent, not only in vitreoretinal surgery but also in endothelial keratoplasty (EK) procedures. The aim of this study was to quantify the direct carbon emissions from the utilization of SF6 in the field of EK over a period of 3 years.

## MATERIALS AND METHODS

This single-center, retrospective case series was carried out at the Royal Liverpool University Hospital, focusing on all endothelial keratoplasties using fluorinated gases. The study spanned 36 months, from January 1, 2021, to January 1, 2024, and all EK procedures—Descemet stripping automated endothelial keratoplasty (DSAEK), Descemet membrane endothelial keratoplasty (DMEK), and EK rebubbling—were included. Data of included procedures were obtained from patients' electronic chart records (MediSight, Medisoft Limited, Leeds, United Kingdom). This study was approved by the Ethics Committee of the Royal Liverpool University Hospital (13016; Ophth/CA/2023-24/12) and was performed in accordance with the tenets of the Declaration of Helsinki.

### Environmental Impact Calculations

The environmental impact of gas tamponade was calculated by converting milliliters (mL) of gas to grams (g) of mass using the modified ideal gas law formula at standard temperature and pressure for the smaller canisters, as described by Moussa et al.^7^ Intraocular gas masses were then converted to their Global Warming Potential over 100 years (GWP100). This study used the GWP100 reference values from the latest IPCC6 assessment report: 25,200 for SF6, 11,100 for C2F6, and 8900 for C3F8.^5^

## RESULTS

In total, 357 procedures requiring anterior chamber (AC) endotamponade with intraocular gases were included. Sulfur hexafluoride diluted with air (SF6 20%) was the only fluorinated endotamponade used, available either in 15-mL (office) or 30-mL (theater) canisters.

### SF6 Canister/Containers Usage

A total of 312 SF6 canisters were used. For procedures performed in theater (n = 296), a 20% solution of SF6 was prepared using 30-mL single-use 100% SF6 canisters (Arcadophta, Toulouse, France). In cases of rebubbling in the office (n = 16), a 20% solution of SF6 was prepared using 15-mL single-use 100% SF6 glass containers (Pharmpur GmbH, Königsbrunn, Germany).

### Number of Primary Surgeries Performed and Type

Between January 1, 2021, and January 1, 2024, a total of 278 EK were performed using fluorinated endotamponades (SF6 30-mL canister). This included 160 DMEK and 118 ultra-thin Descemet stripping automated endothelial keratoplasty (UT-DSAEK) procedures.

Of 160 DMEK surgeries, 71 were standalone DMEK, whereas 79 were triple DMEK (DMEK combined with phacoemulsification and intraocular lens implantation). Among the 118 UT-DSAEK surgeries, 9 cases included an associated phacoemulsification and intraocular lens implantation.

### EK Rebubbling

A total of 79 rebubbling procedures using either filtered ambient air or fluorinated gas were performed. Among DMEK cases, 62 cases (33.7%) underwent graft rebubbling. SF6 gas was used in 38.7% of cases (n = 24), and air was used in 61.3% of cases (n = 38). Notably, multiple rebubblings were required in 5% of cases (n = 8), with SF6 used in half of these cases. Of the 24 DMEK rebubbling procedures using SF6, 16 (66.6%) used SF6 15-mL, with the larger SF6 30-mL canister being used for the remaining 8 rebubblings (33.3%).

Regarding UT-DSAEK, rebubbling was necessary in 12.8% of cases (n = 14), with all rebubbling procedures performed in theater. SF6 gas was used in 57.1% (n = 8) of these cases, whereas air was used in 42.9% (n = 6). Rebubbling had to be repeated in 18.7% of cases (n = 3), with SF6 used in two-thirds (n = 2) of these instances. A summary of all procedures included is presented in Table 1.

**TABLE 1.** Number of Procedures Using Air or Fluorinated Gas at Royal Liverpool University Hospital Between January 2021 and January 2024

Gas	DMEK (%)	DMEK Rebubbling (%)	UT-DSAEK (%)	UT-DSAEK Rebubbling (%)	Total Procedures (%)
Total	160 (100)	62 (100)	118 (100)	17 (100)	357 (100)
SF6 30 mL	160 (100)	8 (12.9)	118 (100)	10 (58.8)	296 (82.9)
SF6 15 mL	0 (0)	16 (25.8)	0 (0)	0 (0)	16 (4.5)
Air	0 (0)	38 (61.3)	0 (0)	7 (41.2)	45 (12.6)

### SF6 Environmental Impact

**FIGURE 1.**
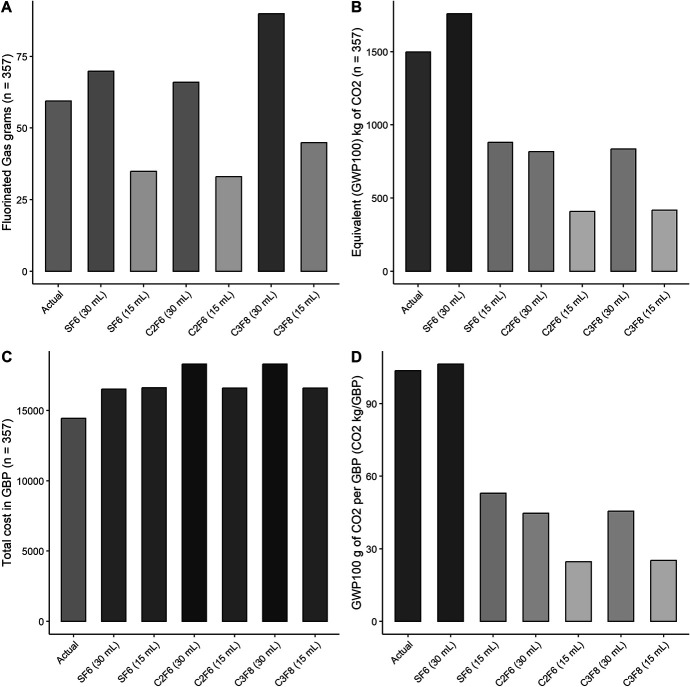
Barplots summarizing the environmental impact of different fluorinated gases' formulations and volumes. Actual values (first bar of each panel) refer to the emissions produced by the sum of all procedures (n = 357) included in this study, whereas the rest of the bars represent the emissions that would be produced assuming that all procedures were performed with the relative fluorinated gas volume and formulation. A, Total mass of fluorinated gas. B, Equivalent total CO_2_ emissions according to the IPCC6 assessment report. C, Total cost in GBP. D, Equivalent CO_2_ emission per GBP.

The 15-mL and 30-mL SF6 canisters used in this case series contain 0.0977 and 0.1955 grams of fluorinated gas, respectively (Fig. 1A). Their combined use over the 3-year study period resulted in an emission equivalent to a mass of almost 1.5 tons of CO_2_ according to the GWP100 conversion (Fig. 1B). This CO_2_ emission is comparable to 13,852.9 km traveled by an average passenger car, using the average emissions of 108.1 g CO_2_/km for new passenger cars in 2022 from the European Environment Agency.^8^

The 30-mL canisters of SF6 contributed the most to the environmental impact of EK included in the study, not only because they were the most frequently used (n = 296) but also because of the higher emission of greenhouse gas per procedure. In fact, the equivalent GWP100 mass of CO_2_ per single canister was 2.463 kg for the 15-mL canister and 4.926 kg for the 30-mL canister. A summary of the environmental impact of the SF6 canisters used in this study is presented in Table 2.

**TABLE 2.** Fluorinated Gas Mass, CO_2_ Mass Equivalent, and Kilometer Equivalent per Procedure by Source

	SF6 15 mL		SF6 30 mL		Total
	Single Canister	n = 16	Single Canister	n = 296	n = 312
SF6 mass per unit (g)	0.0977	1.5632	0.1955	57.868	59.4312
CO_2_ equivalent (kg)	2.463	39.408	4.926	1458.096	1497.504
Passenger car km equivalent	22.8	364.6	45.6	13,488.4	13,852.9

Based on GWP100 values from the Intergovernmental Panel on Climate Change Sixth Assessment Report (2022).

### Comparison With C2F6 and C3F8

Single-use 15-mL and 30-mL canisters of C2F6 and C3F8 are also available in the United Kingdom for use as intraocular endotamponades. When comparing emissions of SF6, C2F6, and C3F8 single-use canisters, the equivalent GWP100 mass of CO_2_ ranges from 1.145 to 4.926, with SF6 30-mL canisters contributing to the emission of greenhouse gases more than 4 times the amount produced by C2F6 or C3F8 single-use 15-mL canisters (Figs. 1A, B).

Prices of single-use canisters are similar across the different fluorinated gases and volumes, ranging from 46.31 to 51.3 British pounds (GBP) (Fig. 1C). Consequently, the GWP100 mass of CO_2_ emitted per GBP by SF6 30-mL canisters was more than 4 times that of C2F6 and C3F8 15-mL canisters and more than double that of single-use SF6 15-mL canisters (Fig. 1D). A summary of the emissions and costs of different fluorinated gases is presented in Table 3.

**TABLE 3.** Comparison of GWP100 CO_2_ Emissions and Cost Among Fluorinated Gasses for Intraocular Endotamponade

	Mass per Unit (g)	CO_2_ Equivalent (kg)	Passenger Car km Equivalent	GBP per Unit	g CO_2_/GBP
SF6					
15-mL canister	0.0977	2.463	22.8	£46.56	52.9
30-mL canister	0.1955	4.926	45.6	£46.31	106.4
C2F6					
15-mL canister	0.0924	1.145	10.6	£46.51	24.6
30-mL canister	0.1847	2.291	21.2	£51.30	44.6
C3F8					
15-mL canister	0.1258	1.169	10.8	£46.51	25.1
30-mL canister	0.2517	2.338	21.6	£51.30	45.6

Based on GWP100 values from the Intergovernmental Panel on Climate Change Sixth Assessment Report (2022).

## DISCUSSION

In this study, the CO_2_ equivalent of fluorinated gases used in EK at a specialized corneal transplant center was assessed, comparing 2 distinct gas delivery systems. This study measures CO_2_ emissions from fluorinated gases in corneal surgery, an area traditionally overlooked environmentally because of the minimal intraocular volumes involved compared with other health care sectors. However, the significant GWP100 of these gases and inefficiencies in certain ocular surgery delivery systems revealed unexpectedly high-carbon emissions. The study found that these emissions could be significantly reduced by using single-use 15-mL borosilicate glass containers, offering a safer and more eco-friendly solution.

The use of intraocular fluorinated gases for the endotamponade of DMEK grafts is widely described and adopted, thanks to the lower risk of graft detachment and need for rebubbling. In their review of 5 studies involving 1195 eyes, Marques et al^9^ suggested that using 20% SF6 over 100% air for AC tamponade in DMEK reduces graft detachment risk. This is attributed to SF6's longer lasting AC bubble and extended postoperative positioning, resulting in 58% fewer rebubbling needed.

Regarding the use of other fluorinated gases as endotamponade in primary surgeries, a large case series of endothelial keratoplasties by Wiley et al^10^ found that the use of 6% C3F8 was superior to that of 20% SF6 in terms of graft detachment rates for DMEK cases, with odds of graft rebubbling in eyes with C3F8 use that were 22% lower than that of SF6. Interestingly, the same study failed to find a similar effect on the detachment rate of eyes undergoing Descemet stripping endothelial keratoplasty. The effect on DMEK detachment rates in Wiley et al might be explained by the longer persistence of C3F8 within the eye compared with SF6, even at concentrations lower than the isoexpansile concentration of 12%.^11,12^ For this reason, the use of C3F8 has also been suggested for repeat rebubbling in cases of failed attempts at rebubbling with air or SF6.^13^

Therefore, some EK procedures at high risk of detachment, and those involving the artificial endothelial layer EndoArt (EyeYon Medical, Israel), may require C3F8 or repeated gas rebubbling.^13,14^ Limited research has explored the clinical efficacy and safety of SF6 as a tamponading agent following donor placement during DSAEK, particularly in comparison with air. Acar et al^15^ demonstrated the comparable effectiveness of SF6 and air in securing the donor graft in Descemet stripping endothelial keratoplasty surgery. Meanwhile, Hyatt et al observed no statistically significant difference in endothelial cell viability between corneal endothelium exposed to SF6 for 10 minutes and unexposed tissue.^16^

The choice of tamponade agents for post-endothelial keratoplasty rebubbling remains a topic of debate. In a study by Keshet et al,^13^ the clinical effectiveness of using C3F8 as a tamponade agent for partially detached DMEK grafts after unsuccessful rebubbling attempts with air or SF6 was investigated. The findings revealed that nonexpansile 10% C3F8 gas successfully secured partially detached DMEK grafts following failed rebubbling attempts with air or SF6.

In this study, we found that the use of single-use smaller canisters of fluorinated gas could significantly reduce the greenhouse gas emissions associated with EK. In particular, 15-mL canisters of C2F6 and C3F8 could represent viable, less polluting alternatives to SF6. Whenever possible, smaller canisters such as 15-mL canisters should be preferred to larger 30-mL canisters. This is also a cost-effective solution, as with our current data we have shown that, with similar costs per procedure, the CO_2_ equivalent emission per GBP was 4 times higher for the 30-mL SF6 canister compared with 15-mL C2F6 or C3F8 and double than that of the 15-mL SF6 canister.

Hypothetically, given the small volumes of intraocular gas needed for EK, manufacturers could consider the production of even smaller single-use canisters for intraocular endotamponade, for instance containing 1 to 5 mL of fluorinated gas, thus reducing the direct emission of greenhouse gas per procedure. However, this benefit would need to be weighed against the increased environmental waste generated by the manufacturing and disposing of single-use canisters containing such a small amount of gas. Small volumes of expansile gas would also be less useful in vitreoretinal procedures, thus reducing the adaptability of such formulations and increasing the risk of waste. Further studies are needed to explore the viability and cost–benefit rate of such adaptations.

Another option to reduce the carbon footprint of corneal surgery, perhaps more easily achievable, could include a policy change that permits the repeated use of a single canister for multiple procedures, assuring the reduction of waste of greenhouse gases. The gas contained in single-use canisters is in fact not sterile, and the use of an air filter during the loading of disposable syringes reduces contaminants. This is therefore not an unrealistic option and is practiced in many countries where variable volumes of gas (eg, 1, 3, or 5 mL) are directly taken for each surgery.^7^ If such a policy were to be applied, it would also open the possibility of using remaining canisters from vitreoretinal surgical lists, which could suit the smaller volumes of endotamponades needed in corneal transplant surgery.

Present data indicate that corneal surgeons should be mindful of the carbon footprint associated with the use of tamponade gases in EK and rebubbling. It is advisable for surgical teams to procure fluorinated gases as needed, minimizing atmospheric waste. Given that SF6, despite being a very effective option for DMEK gas tamponade, also has the highest greenhouse impact among fluorinated gases used in ophthalmic surgery, corneal surgeons might consider more frequent use of air tamponade in selected procedures at lower risk of graft detachment, or lower concentrations of C2F6 or C3F8 as viable alternatives. Over a study period of 3 years of SF6 usage, our results show an environmental impact comparable with that of a car driving 13,852.9 km, and this aspect should be considered when using SF6, especially for rebubbling procedures. Further ecological evaluations will be needed to assess whether the usage of air or a different tamponading agent instead of SF6 could still offset a possible increase in the number of rebubbling procedures.

The aim of this study was to increase awareness within the corneal surgery community about an often-overlooked aspect of their practice. Steps can be taken to lessen the environmental footprint of our health care services while still upholding top-tier safety and care standards for patients. It is crucial for medical practitioners, health care staff, administrative teams, manufacturers, and pharmaceutical firms to recognize the extensive waste produced and its detrimental effects on the environment, public health, long-term health outcomes, and the well-being of future generations.

In conclusion, this study underscores the substantial environmental footprint associated with fluorinated gas use in corneal transplantation procedures. It underscores the potential of 15-mL canisters to mitigate carbon emissions, notwithstanding manufacturing and disposal challenges. These results advocate for an eco-conscious approach, promoting the adoption of smaller, more cost-effective canisters and exploring air as a viable alternative tamponade option when feasible.
